# Wafer-Level Packaging Method for RF MEMS Applications Using Pre-Patterned BCB Polymer

**DOI:** 10.3390/mi9030093

**Published:** 2018-02-25

**Authors:** Zhuhao Gong, Yulong Zhang, Xin Guo, Zewen Liu

**Affiliations:** Institute of Microelectronics, Tsinghua University, Beijing 100084, China; gongzh12@mails.tsinghua.edu.cn (Z.G.); zhangyl15@mails.tsinghua.edu.cn (Y.Z.); guox11@mails.tsinghua.edu.cn (X.G.)

**Keywords:** wafer-level packaging, adhesive bonding, photosensitive BCB, RF MEMS

## Abstract

A radio-frequency micro-electro-mechanical system (RF MEMS) wafer-level packaging (WLP) method using pre-patterned benzo-cyclo-butene (BCB) polymers with a high-resistivity silicon cap is proposed to achieve high bonding quality and excellent RF performance. In this process, the BCB polymer was pre-defined to form the sealing ring and bonding layer by the spin-coating and patterning of photosensitive BCB before the cavity formation. During anisotropic wet etching of the silicon wafer to generate the housing cavity, the BCB sealing ring was protected by a sputtered Cr/Au (chromium/gold) layer. The average measured thickness of the BCB layer was 5.9 μm. In contrast to the conventional methods of spin-coating BCB after fabricating cavities, the pre-patterned BCB method presented BCB bonding layers with better quality on severe topography surfaces in terms of increased uniformity of thickness and better surface flatness. The observation of the bonded layer showed that no void or gap formed on the protruding coplanar waveguide (CPW) lines. A shear strength test was experimentally implemented as a function of the BCB widths in the range of 100–400 μm. The average shear strength of the packaged device was higher than 21.58 MPa. A RF MEMS switch was successfully packaged using this process with a negligible impact on the microwave characteristics and a significant improvement in the lifetime from below 10 million to over 1 billion. The measured insertion loss of the packaged RF MEMS switch was 0.779 dB and the insertion loss deterioration caused by the package structure was less than 0.2 dB at 30 GHz.

## 1. Introduction

Radio-frequency micro-electro-mechanical system (RF MEMS) devices, such as switches, tunable capacitors and resonators, have movable and fragile parts that must be protected from damage and contamination during wafer handling and dicing [1,2,3]. Besides, packages of the defective MEMS result in a higher cost due to subsequent low yields and extra steps in the process. Furthermore, the MEMS devices have strict requirements on the level of gas leakage and ambient pressure associated with gas composition inside the encapsulation [4,5]. For instance, the MEMS metal-contact switches are sensitive to moisture and particles from ambient environment, as the contamination of the contacts has a detrimental effect on the switching reliability. Therefore, the package must offer encapsulation with a low gas leak rate in an inert ambient gas, such as nitrogen, at atmospheric pressure to ensure the long-term stability and reliability of the MEMS switches. To solve these problems, the packaging is preferably implemented on a wafer level prior to die singulation, which is known as the wafer-level package method [6,7].

There are generally two types of bonding technology used in wafer-level packaging (WLP) for RF MEMS applications: anodic bonding and intermediate layer bonding. The anodic bonding technique can provide a strong bond and hermetic seals but suffers from the requirements of a relatively high temperature (typically 400 °C), high voltage (0.5 to 1 kV) as well as a flat and particle-free surface [8]. The intermediate layer bonding process, such as eutectic bonding [9], gold-gold thermo-compression bonding [10], glass frit bonding [11] and adhesive bonding [12,13], can accommodate topography and particles on wafer. Nevertheless, the sealing ring is made of conductive materials for the metal intermediate layer bonding, buried feedthroughs or vertical interconnections have to be fabricated through a complex vertical through-via process. By contrast, the electrical connection process of the adhesive wafer bonding using polymer materials as bonding layers is relatively simple as signal feedthroughs can be implemented in a straightforward manner by passing through the electrical insulating polymers. Besides, other advantages of the adhesive bonding method over other bonding methods include good compensation of surface patterns and contamination of particles, low processing temperature and simple patterning process. One of the promising candidates of polymer adhesives for RF MEMS applications is benzo-cyclo-butene (BCB) from Dow Chemical Company. BCB is an organic resin with superior electrical, mechanical and thermal properties due to its high resistivity (10^19^ Ω·cm), low loss tangent (0.0008–0.002), low permittivity (2.65), low moisture absorption (<0.2%), low out-gassing, low cure temperature (<250 °C), small volume shrinkage during curing (5–6%) and excellent dielectric stability over a wide frequency range [14,15,16]. Additionally, BCB as the intermediate bonding material offers the highest bond strength in comparison to other conventional bonding polymers [17].

According to the conventional bonding process [3], the BCB layer is spin-coated on the side with the cavities and patterned by dry-etching or by using a photosensitive version of the material to form sealing rings after the etching of the housing cavities. However, the liquid-like BCB tends to flow down from sharp topography edges, which creates a massive problem. In this paper, a new approach for the wafer-level bonding based on a pre-patterned BCB process is proposed to offer a fine bonding interface on a severe topography surface for excellent package quality. The package design is simulated and analyzed first in Section 2. The proposed packaging process is described and compared with the conventional processes in Section 3. After this, a comprehensive test of the package properties is conducted, including bonding quality, RF performance and reliability in Section 4. Finally, Section 5 concludes this paper.

## 2. Package Design

In the package design of RF MEMS applications, there are many factors that should be considered, such as packaging materials, electrical connections, sealing width, cavity height, compatibility with other processes and so on. The coplanar waveguide (CPW) line, the most common RF feedthrough method, is fabricated on the device wafer in order to evaluate the effect of the package on RF performances of RF MEMS devices. To form the CPW line with minimum insertion loss, the Au layer is electroplated on the Borofloat glass (BF33) wafer with excellent insulation performance. Furthermore, the transparency property of glass makes it possible to inspect the bonding quality through a microscope. Photosensitive BCB CYCLOTENE 4024–40 [18] is used as a patternable bonding and sealing material with a designed layer thickness of over 5 μm to compensate the Au CPW structure with about 2 μm in vertical height. For the cap wafer material, the resistivity is the key point as the RF performance of packaged device deteriorates as a result of the proximity effect induced by the cap on the MEMS surface [19]. High resistivity silicon (HR_Si) with a resistivity of over 1000 Ω·cm is chosen as the cap wafer material to diminish the proximity effect and reduce the attenuation of the package.

**Figure 1:**
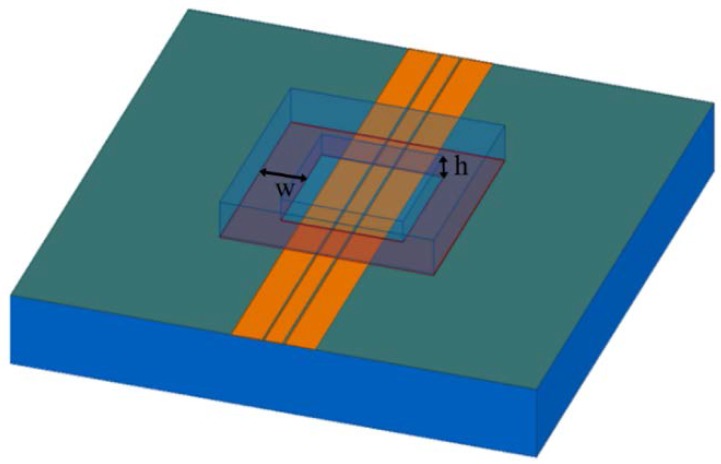
Simulation model of the proposed package.

**Figure 2:**
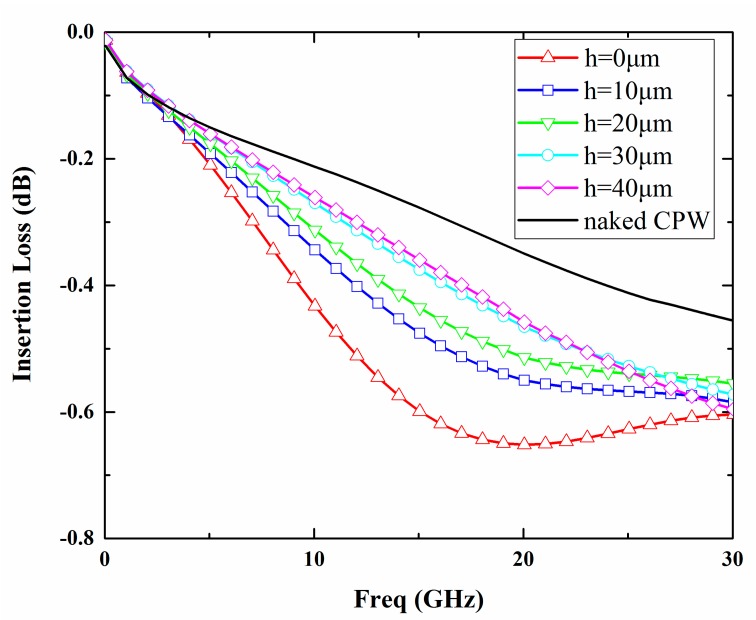
Simulated insertion loss of the naked coplanar waveguide (CPW) and packaged CPW with different cavity heights (sealing width is fixed to 300 μm).

**Figure 3:**
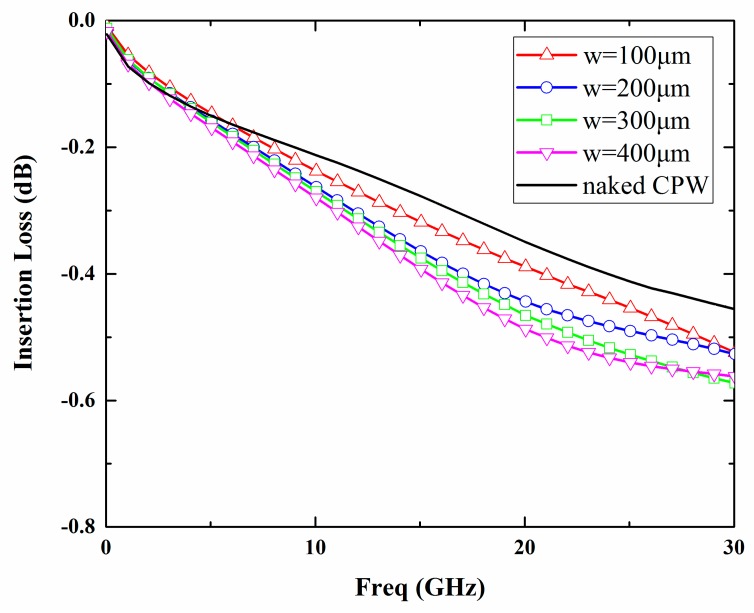
Simulated insertion loss of the naked CPW and packaged CPW with different sealing widths (cavity height is fixed to 30 μm).

The width of the signal line and the spacing between the signal line and ground line are designed to be 120 μm and 16 μm respectively so that the characteristic impedance of the 3500 μm long CPW line is 50 Ω. In addition, the influence of the package dimensions on the RF characteristics is analyzed with a commercial simulation tool, Ansoft high-frequency structure simulator (HFSS). A schematic of the simulation model is depicted in Figure 1. The simulated insertion losses of the package for 0–30 GHz based on different cavity heights (h) and sealing widths (w) are shown in Figure 2 and Figure 3. It can be seen from the simulation results that with an increase in the cavity height from 0 to 30 μm, the insertion loss of the package decreases and approaches that of the naked CPW. However, if the cavity continues to increase, the insertion loss almost maintains the same level. In order to minimize the insertion loss of the packaged CPW, a cavity height of over 30 μm is needed. According to the variations of sealing widths, the insertion loss decreases slightly as the sealing width decreases due to the low dielectric constant of the BCB polymer. Taking into account the cost of the silicon deep etching process and RF performance, the best height of the cavity was determined to be 30 µm, while BCB sealing width was changed to be in the range of 100–400 μm in order to investigate its influence on shear strength of the proposed package.

## 3. Packaging Process and Comparison with the Conventional Process

**Figure 4:**
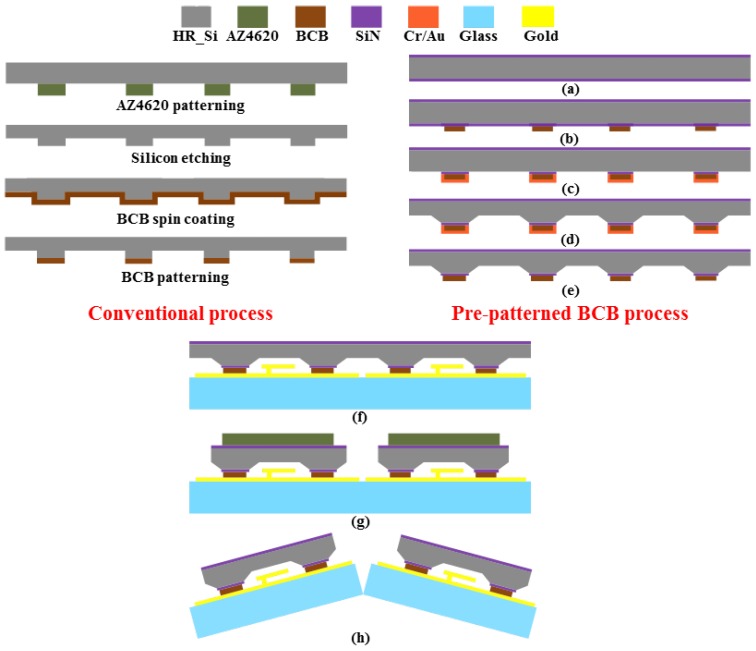
The conventional and the proposed (pre-patterned benzo-cyclo-butene (BCB)) packaging process.

As shown in Figure 4, a conventional packaging process and the proposed packaging process based on pre-patterned BCB technology aimed at improving bonding quality have been developed and compared by studying the flatness and uniformity characteristics of the BCB layer coated over the wafer with deep cavities. In the conventional process, photoresist (AZ4620) with a thickness of 8 μm was first patterned as a mask layer for dry etching of silicon on the cap wafer for housing cavities. After this, the BCB is spin-coated and patterned onto the bonding area to form sealing rings, with the experimental conditions of the BCB patterning given in Table 1. However, for a wafer with severe topography, including deep recesses or high steps, the main problem has been that the coated polymer tends to flow down the sharp topography edge, which results in a non-flat surface due to its liquid-like behavior. In addition, it is difficult to achieve the BCB film with uniform thickness over this type of surface. These problems have a negative impact on the wafer bonding quality and might result in the final packaged RF MEMS device not working properly.

In order to obtain a BCB layer with uniform thickness and a flat surface, which plays an important role in achieving a robust package with excellent bonding quality, an improved packaging process is proposed. This process is based on pre-patterned BCB, which means that the BCB polymer is patterned before the etching of the housing cavity in the cap wafer to form the sealing ring and the bonding layer. Packaging experiments are carried out in four-inch (100) oriented p-type high resistivity silicon wafer with a thickness of 500 μm. Figure 4 shows the details of the fabrication process flow:

(a) A layer of 50-nm thick silicon nitride (Si_3_N_4_) is deposited as the silicon etching mask by low pressure chemical vapor deposition (LPCVD) on both sides of the silicon cap wafer.
(b) BCB is spin-coated at 2000 rpm (rounds per minute) and patterned through standard photolithography with UV (Ultraviolet Rays) lights.
(c) The Cr and Au layer is sputtered successively on the cavity side of the wafer to form the adhesion layer and the hard mask layer to protect the BCB during the silicon etching. After this, the Cr/Au layer and Si_3_N_4_ layer are patterned to create the opening for the following cavity etching.
(d) Tetramethyl ammonium hydroxide (TMAH) anisotropic etching is used to manufacture the cavities on the cap wafer.
(e) The pre-patterned BCB process on the cap wafer is finished after the removal of the Cr/Au layer with the corresponding solution.
(f) The cap wafer is bonded to the device glass wafer with CPW structures in a wafer level.
(g) AZ4620 with a thickness of 8 μm is spin-coated and patterned to define the areas on the cap wafer above the electrical pads of the device wafer. After this, the cap wafer is partially diced to provide access to the electrical pads by adjusting the height of wafer dicing saw to the device wafer.
(h) Finally, the device wafer is diced to yield individual 0-level packaged RF MEMS devices.

The silicon cavity is formed through the wet etching technique with TMAH solution because the dry etching process, such as DRIE (deep reactive ion etching), emits a great deal of heat. Subsequently, the BCB polymer would be cross-linked to a certain degree before the bonding, resulting in lower bonding strength and higher gas leakage. The etching rate of the TMAH anisotropic etching increases with increasing process temperature and decreasing solution concentration. At 70 °C, the etching rate is measured as 0.3 μm/min for the 5 wt % TMAH solution with a surface roughness of 45.37 nm. Thus, the silicon wafer is wet-etched under this condition for 100 min to achieve the demanded housing cavities with a depth of 30 μm.

Among the different types of mask material (e.g., photoresist, Si_3_N_4_ and metal), sputtered Cr/Au is used as the adhesion and hard mask layers to protect the BCB during the wet etching process due to its superior features:

(1) Low processing temperature compared to the chemical vapor deposition of Si_3_N_4_ or SiO_2_.
(2) Chemically resistant enough for the wet-etching process as the polymer would react with the alkaline solution, which makes it unsuitable to serve as the mask layer in TMAH.
(3) Excellent step coverage ability: the sputtering method can provide a good coverage for the side wall of the BCB sealing ring.
(4) Process compatibility with BCB: Au and Cr can be removed successively by potassium iodide (KI) and diluted nitric acid (HNO_3_) with no effect on the BCB polymer.

The thickness of the Au mask layer is also a parameter to consider. A thicker metal layer means more stress, which might lead to the deformation of the basically liquid BCB layer. To avoid the surface voids on the cap wafer caused by this deformation, the gold layer thickness should be less than 150 nm according to the observation of BCB surfaces sputtered by gold with different thickness. Consequently, the thicknesses of Cr and Au are designed to be 5 nm and 100 nm, respectively.

**Figure 5:**
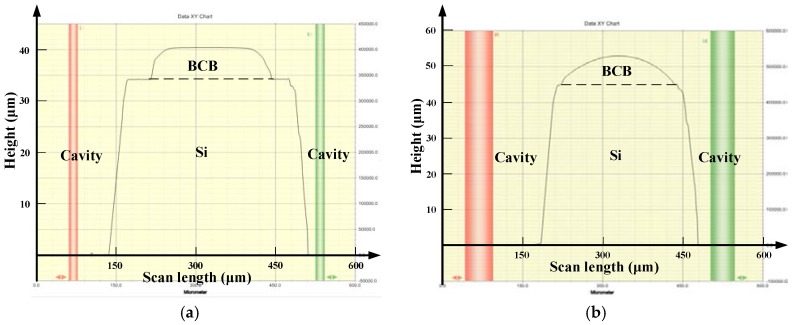
Measured BCB sealing ring height profile of (**a**) the pre-patterned BCB process and (**b**) the conventional process.

**Figure 6:**
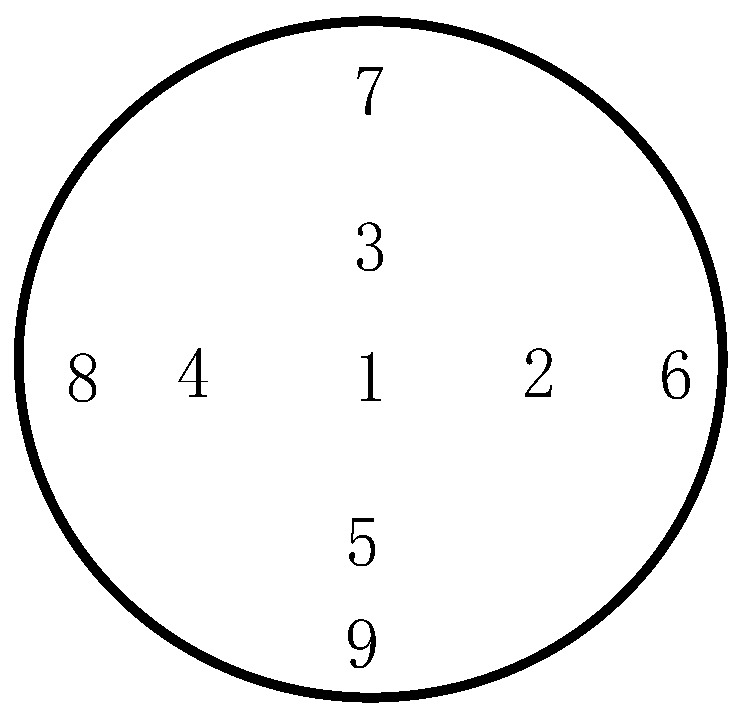
Schematic diagram of measurement point positions on the wafer.

After silicon wet-etching for the cavity, the achieved height profile of the flat BCB sealing ring is presented in Figure 5a using a surface profiler (Dektak150, Veeco, Plainview, NY, USA). As a comparison, Figure 5b shows that the non-flat BCB ring profile is similar to the parabolic shape of the conventional process. For the conventional process, since BCB is spun over a surface with strong topography (>10 μm in height), the height in the center of the BCB ring is larger than that in the periphery due to its high liquidity. The gravity reducing the film thickness on the edge and surface tension of the polymer pulling the polymer back from the corner are both responsible for the combined effect [20]. Furthermore, the thicknesses of BCB layers were measured over the whole wafer for these two packaging methods and concluded in Table 2, according to the different measurement point positions provided in Figure 6. Table 2 also contains the respective calculated average value (AVG) and mean square error (σ) of the BCB thickness. The BCB film obtained by the proposed process has a uniform thickness with a mean square error of 0.2016 μm. However, the σ value for the conventional process is 0.8497 μm, which is over four times higher than the former one, showing that a great improvement could be made in the uniformity of the BCB film by adopting the pre-patterned BCB process.

To form the CPW lines on the device wafer, a Ti adhesion layer with a thickness of 20 nm and Au seed layer with a thickness of 200 nm are sequentially sputtered on the device wafer. After this, an Au layer with a thickness of 1.8 µm is selectively electroplated through the photoresistant (AZ4620) mold. The width of the CPW line is 120 µm and the spacing between the CPW lines is 16 µm as previously designed to achieve the characteristic impedance of 50 Ω.

**Figure 7:**
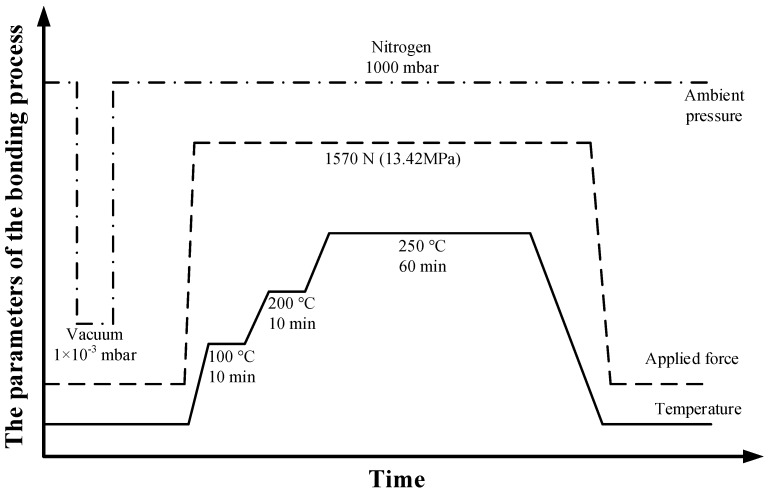
The parameters of the bonding process.

**Figure 8:**
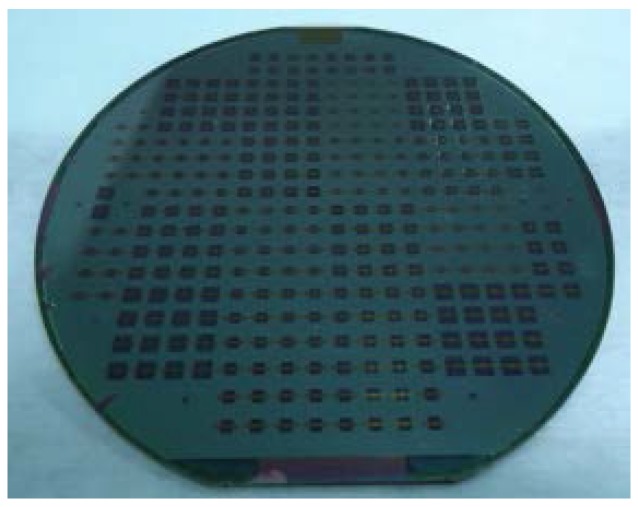
Photograph of the wafer-level bonded package.

**Figure 9:**
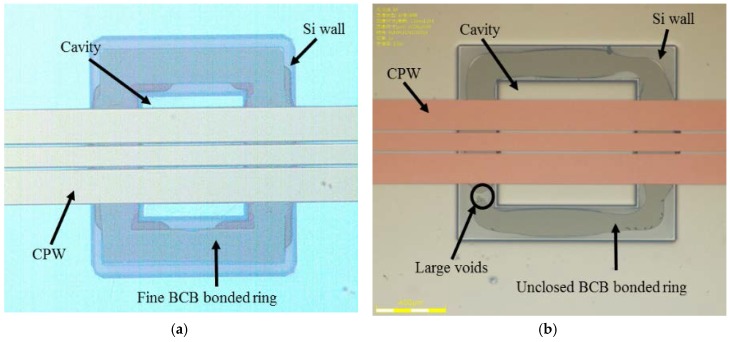
Microscope images of the bonded BCB interface from the back side for: (**a**) the pre-patterned BCB process and (**b**) the conventional process.

The BCB-coated cap wafer is submitted to a soft bake process (120 °C for 20 min) to remove gases and water, which might be absorbed on the bonding surfaces before the bonding procedure. After the baking process, two wafers are aligned with the SUSS MA6 mask aligner and brought into contact in a wafer bonder (CB6L, SUSS MicroTec, Garching, Germany). The parameters of the bonding process are given in Figure 7. The bonding is conducted with a static force of 1570 Nin a nitrogenous atmosphere at a standard atmospheric pressure (1000 mbar). The total bond area is ∼1.17 × 10^−4^ m^2^, thus resulting in an effective bonding pressure of 13.42 MPa. Following this, the bonded wafer is heated to a peak temperature of 250 °C with a dwelling time of 1 h. During this step, the BCB is fully cured and forms a continuous layer to ensure a strong bonding between two wafers. The wafer-level bonded package is shown in Figure 8. The backside of the CPW line and the patterned BCB ring around the CPW line can be seen through the transparent glass substrate using an optical microscope after bonding. As shown in Figure 9a, no gaps or voids are found on the bonded area of rectangular ring shape, which is consistent with the BCB pattern before packaging. This suggests that the BCB layer has good contact with both the cap and device wafers, conforming well to the bonding surfaces with protruding metal lines. For the sample packaged in the conventional process, the BCB bonded area with defects and voids at the joint interface is shown in Figure 9b. The unbonded area is generally located on the periphery of the ring, which indicates the source of the voids to be the parabolical shaped surface and non-uniformity of the thickness of the BCB layer. Moreover, the BCB bonded ring is not closed, which corresponds to a non-hermetic package that could not meet the requirements for the RF MEMS applications. In summary, the proposed packaging process has advantages in the surface flatness, thickness uniformity and bonded interface quality of the BCB layer over the traditional process by adopting the method of patterning the BCB film before the Si cavity etching. Therefore, a sealing result of the packaging process with high bonding strength is expected.

**Figure 10:**
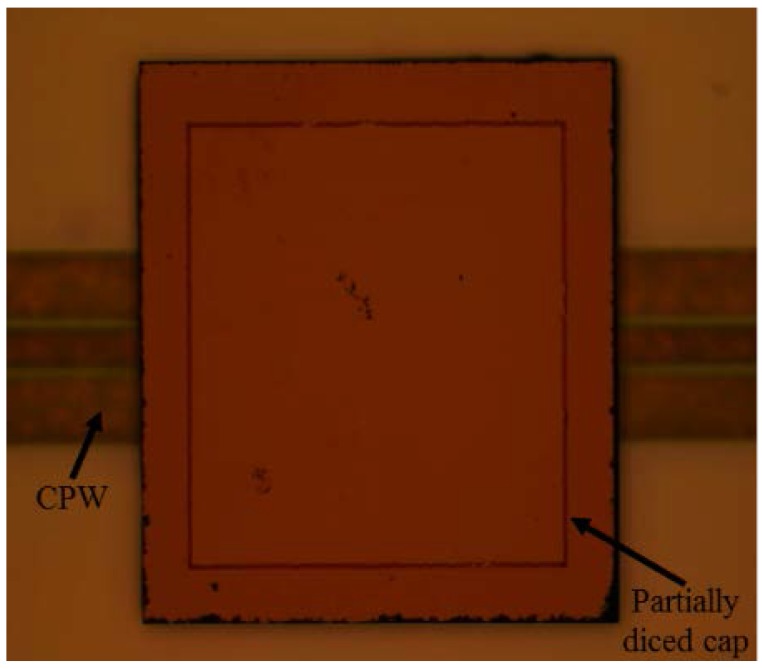
Microscope image of the packaged CPW on the substrate wafer after dicing the cap wafer.

In order to provide access to the contact pads, the cap above the pads should be removed by the partial dicing of the cap wafer, which is shown in Figure 10. During this dicing process, the height of the diamond dicing saw needs to be carefully adjusted so that it could dice the cap wafer thoroughly without hitting the device wafer. In addition, the bonding strength of the proposed packaging is proven to be strong enough to withstand the dicing process and achieve a high yield of 90% as the packaging cap does not fall off in scribing. Finally, the bonded wafer is diced into a unit cell of which the size is 4 × 4 × 1 mm^3^.

## 4. Results and Discussions

To evaluate the properties of the wafer-level packaging comprehensively, several tests and measurements are performed, including scanning electron microscope (SEM) observation and a shear strength test with different BCB widths for bonding quality. For further investigation of the influence of the packaging process in terms of the RF signal transmission characteristics and lifetime of the RF MEMS device, a RF MEMS switch is packaged in the proposed approach.

### 4.1. Bonding Quality

**Figure 11:**
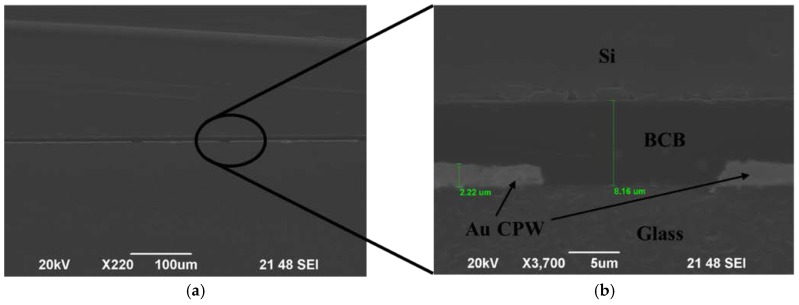
SEM photographs of cross-sectional view on the CPW after bonding: (**a**) overview and (**b**) enlarged view of the bonding interface.

The cross-sectional observation of the bonding interface is carried out to visually assess the bonding quality of the BCB polymer with a scanning electron microscope, which is shown in Figure 11. In Figure 11b, the details of the BCB areas and the CPW lines between two bonding wafers are presented, showing no voids in the BCB layer or at the bonding interface. The gold conducting lines are measured as having a thickness of 2.2 μm and are perfectly embedded in the BCB, which implies a robust package. This also shows that the BCB layer thickness is reduced to be about 5.9 µm after the bonding process.

To illustrate the bonding strength of the BCB bonding process, the shear strengths of packaged samples are tested with the DAGE series 4000 Bondtester. In this paper, the bonding pattern is a closed rectangular sealing ring with an inner size of 836 × 852 μm^2^ with BCB widths of 100, 200, 300 and 400 µm, respectively. Since the package structure is not completely symmetrical, the shear strength is tested in two directions: parallel and vertical to the CPW line. The average results of shear force test for five samples under different conditions are summarized in Table 3. The shear strengths are calculated according to the corresponding bonding areas for different BCB widths. This found that the shear force increases with increasing BCB width, while the shear strength decreases with increasing BCB width. On the other hand, the shear strength of the package tested in the direction vertical to the CPW line is higher than that parallel to the CPW line. This is because additional force is required to get across the 2.2-μm thick CPW lines in case the capping chip is sheared off vertically. According to the military standard (MIL-STD)-883E method 2019.5, a shear strength of over 6 MPa is required to ensure the bonding quality of the packaged devices with bonding areas smaller than 4 mm^2^. The measured results in the range of 21.58–54.74 MPa show that the shear strength of the package is well above the MIL-STD-883E specifications.

### 4.2. RF Characterization and Reliability of Packaged RF MEMS Switch

**Figure 12:**
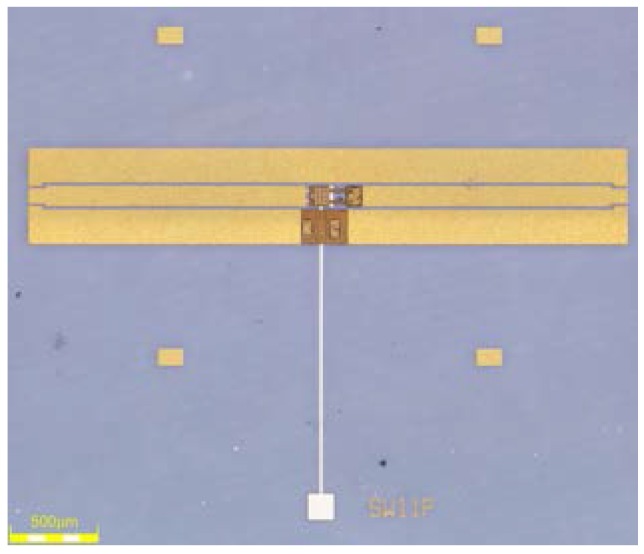
Photograph of the radio-frequency micro-electro-mechanical system (RF MEMS) switch for packaging.

To illustrate the feasibility of this packaging method and to explore the impact of this process on the RF and reliability performance of the RF MEMS devices with movable structures, the application of this approach for a RF MEMS switch was implemented and tested. A series of high performance RF MEMS switches with Au-Au contacts, which were described in an earlier work [21], are packaged through the pre-patterned BCB packaging process in a wafer level. Figure 12 shows this series switch consisting of a movable gold cantilever, which is suspended above the actuation plate and mechanically anchored to the signal line of the CPW.

**Figure 13:**
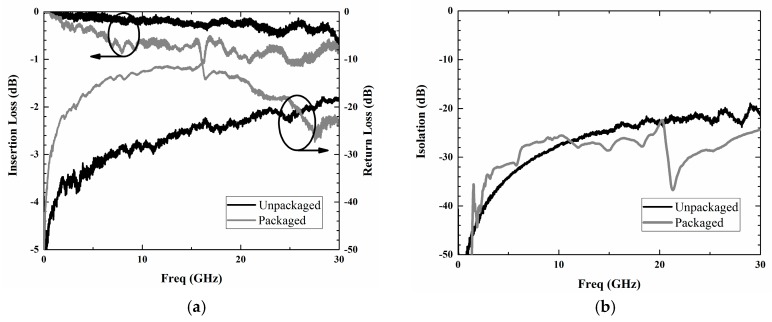
RF characteristics of the fabricated RF MEMS switch before and after packaging: (**a**) insertion loss and return loss and (**b**) isolation.

The RF characteristics of the unpackaged and packaged RF MEMS switch in the frequency range from dc to 30 GHz were measured using E8363C vector network analyzer (Agilent, Santa Clara, CA, USA) with Summit 12000M probe station and on-wafer SOLT (short-open-load-through) calibration standard, which is shown in Figure 13. The measured insertion loss and isolation of the switch before packaging were 0.599 dB and 21.03 dB at 30 GHz, respectively. The return loss was measured to be less than 18.7 dB from dc to 30 GHz. After the measurement of the bare switch, the cap wafer on which the BCB sealing rings and housing cavities formed becomes bonded to the device wafer with the BCB width of 300 μm. Following this, the electrical pad access is obtained by optional dicing for the following RF performance measurement. The measured insertion loss and isolation of the packaged switch were 0.779 dB and 23.07 dB at 30 GHz, respectively. From these results, the extra insertion loss caused by the package was 0.18 dB at 30 GHz. In addition, the isolation of the packaged switch was higher than that of the unpackaged one, which is most likely due to a temperature-induced curvature of the released cantilever [22,23]. However, the return loss of the package switch is relatively low with the minimum value of about 12 dB, which indicates the poor matching performance of the whole structure. This mismatch is attributed mainly to the decrease in the characteristic impedance for the CPW line under the BCB ring. Therefore, the matching performance of the RF MEMS switch could be improved by redesigning the dimension of CPW under the BCB ring to achieve the characteristic impedance of 50 Ω.

**Figure 14:**
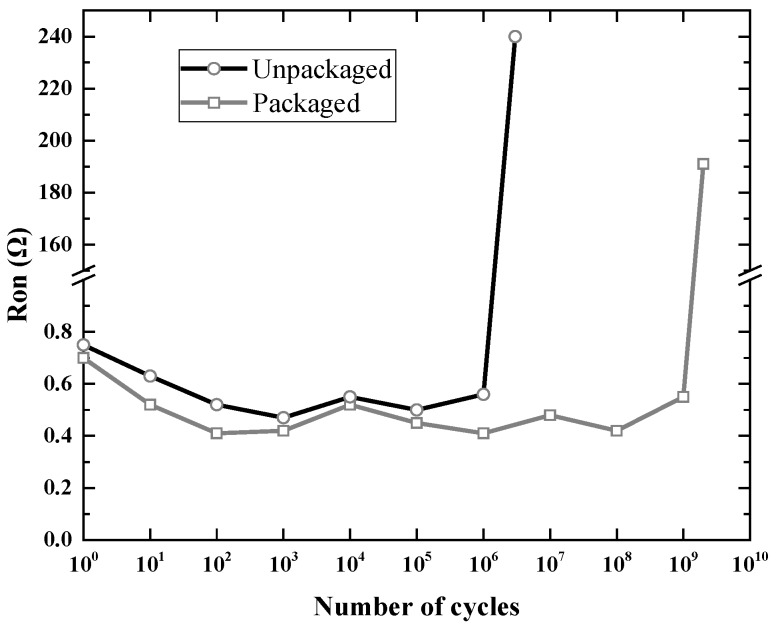
The measured contact resistance as a function of the number of switching cycles for the packaged and unpackaged RF MEMS switch.

The contact resistance is measured with a Keithley 2110 multimeter as a function of the number of switching cycles to evaluate the effect of the package on the reliability of the RF MEMS switches. The reliability measurement is performed at a switching rate of 10 kHz with the actuation voltage of 40 V. During the experiment, an oscilloscope is used to monitor the voltage on the switch contact to make sure that the contact resistance does not change drastically. Five unpackaged switches and three packaged switches were tested in this reliability measurement method. We observed that all of the unpackaged switches fail within 10 million switching cycles in air, while the cycling lifetime of packaged switches with a nitrogen atmosphere in the cavity was higher than 1 billion. A typical measurement result of the unpackaged and packaged switch is shown in Figure 14. The unpackaged switch works properly in air for 3 × 10^6^ switching cycles until the contact resistance increases to the order of 100 Ω. In contrast, the packaged switches show significantly improved reliability and lifetime of about 2 billion cycles. This indicates the effectiveness of the package as a barrier to moisture and particles from ambient environment as organic contaminants lead to immediate device failure.

## 5. Conclusions

In this paper, a wafer-level packaging method based on the BCB polymer for RF MEMS applications has been introduced. To achieve high quality for the bonding interface to the cap wafer with a severe topography surface, the BCB sealing ring is patterned prior to the formation of housing cavities. During the wet etching of silicon, the patterned BCB layer is covered with the Cr/Au layer to protect the BCB from the damage of TMAH solution. Compared with the conventional packaging process, this process results in the BCB layer with better surface flatness and thickness uniformity, which is more suitable for wafer bonding. After bonding of the cap wafer to the device wafer with CPW lines, the shear strength test is implemented with the BCB sealing width ranging from 100 μm to 400 μm. Shear force improves with increasing ring width, while shear strength decreases with increasing ring width. The shear strength of the packaged devices is higher than 21.58 MPa, showing that the bonding quality satisfies the requirement of MIL-STD-883E. To illustrate the feasibility of this process and to measure the RF characteristics and reliability of the RF MEMS device in this package, a RF MEMS switch is packaged using this approach. The insertion loss and return loss of the packaged switch are 0.779 dB and 23.07 dB at 30 GHz with a high lifetime of over 1 billion cycles. In conclusion, we demonstrate that a strong and reliable RF MEMS package with low loss can be realized using the pre-patterned BCB technique.

## Figures and Tables

**Table 1:** The process conditions of the BCB patterning.

Adhesion Promoter (AP3000 ^a^)	3000 rpm, 20 s
BCB (4024-40) spin coating	2000 rpm, 30 s
Soft bake	70 °C, 90 s
Expose	210 mJ/cm^2^
Pre-develop bake	60 °C, 60 s
Immersion develop (DS3000 ^a^)	38 °C, 200 s
Post-develop bake	90 °C, 60 s

^a^ From Dow Chemical Company.

**Table 2:** The thicknesses of BCB layers over the wafer.

Measurement Points	1	2	3	4	5	6	7	8	9	Avg	σ
Pre-patterned BCB process (μm)	7.29	6.72	7.00	7.18	7.36	6.96	6.93	7.22	7.10	7.08	0.2016
Conventional process (μm)	10.32	9.18	8.50	9.53	8.07	8.34	8.28	7.62	8.12	8.66	0.8497

**Table 3:** Summary of shear force and shear strength results.

BCB Width (μm)	Bonding Area (mm^2^)	Parallel to the CPW Line		Vertical to the CPW Line	
		Shear Force (kgf)	Shear Strength (MPa)	Shear Force (kgf)	Shear Strength (MPa)
100	0.3776	1.637	42.49	2.109	54.74
200	0.8352	2.265	26.58	2.881	33.80
300	1.3728	3.265	23.31	4.199	29.98
400	1.9904	4.383	21.58	4.993	24.58
